# Polydopamine-Coated Copper-Doped Co_3_O_4_ Nanosheets Rich in Oxygen Vacancy on Titanium and Multimodal Synergistic Antibacterial Study

**DOI:** 10.3390/ma17092019

**Published:** 2024-04-26

**Authors:** Jinteng Qi, Miao Yu, Yi Liu, Junting Zhang, Xinyi Li, Zhuo Ma, Tiedong Sun, Shaoqin Liu, Yunfeng Qiu

**Affiliations:** 1 College of Chemistry, Chemical Engineering and Resource Utilization, Northeast Forestry University, Harbin 150040, China; gentry7321@163.com; 2 Key Laboratory of Microsystems and Microstructures Manufacturing, School of Medicine and Health, Harbin Institute of Technology, Harbin 150080, Chinashaoqinliu@hit.edu.cn (S.L.); 3 School of Life Science and Technology, Harbin Institute of Technology, Harbin 150001, China

**Keywords:** medical titanium, antibacterial nanocoatings, oxygen vacancies, oxidative stress, multimodal synergy

## Abstract

Medical titanium-based (Ti-based) implants in the human body are prone to infection by pathogenic bacteria, leading to implantation failure. Constructing antibacterial nanocoatings on Ti-based implants is one of the most effective strategies to solve bacterial contamination. However, single antibacterial function was not sufficient to efficiently kill bacteria, and it is necessary to develop multifunctional antibacterial methods. This study modifies medical Ti foils with Cu-doped Co_3_O_4_ rich in oxygen vacancies, and improves their biocompatibility by polydopamine (PDA/Cu-O_v_-Co_3_O_4_). Under near-infrared (NIR) irradiation, nanocoatings can generate •OH and ^1^O_2_ due to Cu^+^ Fenton-like activity and a photodynamic effect of Cu-O_v_-Co_3_O_4_, and the total reactive oxygen species (ROS) content inside bacteria significantly increases, causing oxidative stress of bacteria. Further experiments prove that the photothermal process enhances the bacterial membrane permeability, allowing the invasion of ROS and metal ions, as well as the protein leakage. Moreover, PDA/Cu-O_v_-Co_3_O_4_ can downregulate ATP levels and further reduce bacterial metabolic activity after irradiation. This coating exhibits sterilization ability against both *Escherichia coli* and *Staphylococcus aureus* with an antibacterial rate of ca. 100%, significantly higher than that of bare medical Ti foils (ca. 0%). Therefore, multifunctional synergistic antibacterial nanocoating will be a promising strategy for preventing bacterial contamination on medical Ti-based implants.

## 1. Introduction

Bacterial infection has become a critical threat to global human health, which needs to be urgently addressed. The hazard of bacterial infections from implantable medical devices during clinical treatment is quite difficult to avoid [1], and can even be directly described as a fatal threat to patients. Medical titanium (Ti) and its alloys have been widely recognized for the clinical application of implants in orthopedics and dentistry due to their considerable chemical inertness, favorable mechanical properties, and excellent biocompatibility [2,3,4], but the problem of bacterial infections associated with their pre- and post-operative periods threatens their successful operation to a great extent, and the infections frequently manifest themselves in the localized pain and postponed healing [5], which can cause additional disturbances and economic losses to the patients and their families. In the past, the utilization of antibiotics for the treatment of bacterial infections arising from implants has obtained satisfactory results, but the abuse of antibiotics has also caused a variety of negative effects, such as the emergence of bacterial drug resistance and rapid bacterial evolution and mutation, etc., which have significantly diminished the efficacy of antibiotics [6,7]. Therefore, it is necessary to develop and innovate multifunctional nanomaterial platforms with higher efficiency and controllability to compensate for antibiotics.

In this regard, multifunctional nanomaterials’ platform has drawn tremendous attention in recent years to defend against pathogenic bacterial infections, owing to their distinctive physicochemical properties including a quantum size effect and high specific surface area, superior antibacterial efficiency, lower bacterial drug resistance, and biocompatibility [2,8]. The nanomaterials’ modification of Ti implants showed promising potential for improving the antibacterial performance. For instance, polydopamine (PDA) was able to form a stable and thin nanofilm on Ti implants, which could further coordinate with metal ions or organic compounds to prepare a multifunctional antibacterial layer [9,10]. Yiwen Li recently summarized progress of PDA-based antibacterial composites, indicating their convenient process and efficient activity for fabricating PDA composite antibacterial interfaces [11].

It is worth noting that Co-based nanomaterials have proven to be promising candidates for sterilization. For example, Li et al. found that a Co_3_O_4_ nanowire electrode was capable of discharging low voltage electricity to effectively prevent bacterial skin infections [12]. Tian et al. developed a hierarchical macro/mesoporous Co_3_O_4_-SiO_2_, which could eradicate the antibiotic-resistant bacteria [13]. The porous Co_3_O_4_ nanoplates possessed synergetic photothermal/photodynamic therapy through a DNA damage route [14]. Thus, applying Co_3_O_4_-based composites on Ti-based implants will be an alternative way to suppress bacterial infection. In addition, Cu is a perspective inorganic antibacterial agent and Cu-based nanocoatings can potentially combat bacterial adhesion as well as prevent the formation of biofilms on implantable medical devices [15]. For instance, the minimum bactericidal concentration (MBC) of Cu^2+^ against *S. aureus* was 7.04 μg/mL [16]. Ning et al. found that the MBC of Cu^2+^ against *S. aureus* was only 1.6 μg/mL [17]. Zhao et al. found that Cu_2_O-TiO_2_/Ti_2_O_3_/TiO exhibited the contact sterilization property [18]. Recently, Wang’s work proved that Cu^2+^ sterilization and contact sterilization were involved when 0.034 μg/mL of Cu^2+^ was released [19]. Inspired by these findings, Cu-doped Co_3_O_4_ is assumed to display enhanced Fenton-like activity due to the synergetic Co−Cu electronic interaction [20]. However, few papers on Cu-doped Co_3_O_4_-based antibacterial nanocoating on Ti-based implants were reported.

In this work, PDA/Cu-O_v_-Co_3_O_4_ was designed and synthesized by electrochemical deposition, alkaline oxidation polymerization, and plasma-enhanced chemical vapor deposition (PECVD) methods. The presence of PDA coating enhanced its biocompatibility and photothermal property. Herein, PECVD was an effective strategy to modulate the micro-/nanostructures and composition of Cu-O_v_-Co_3_O_4_, such as pores, oxygen defects, etc., which was regarded to be a potential factor for modulating the physicochemical properties. Co_3_O_4_, O_v_-Co_3_O_4_, and Cu-O_v_-Co_3_O_4_ were also prepared by similar methods. The antibacterial property of these materials to Gram-negative *Escherichia coli* (*E. coli*) and the Gram-positive *Staphylococcus aureus* (*S. aureus*) was examined under 808 nm of irradiation and compared. It was found that PDA/Cu-O_v_-Co_3_O_4_ showed synergistic effects for sterilization including a metal ion release, Fenton-like reaction, and photothermal and photodynamic effect compared to these control groups. A systematic study disclosed that the ATP and protein levels in bacteria were downregulated, and the membrane permeability was increased after treatment by PDA/Cu-O_v_-Co_3_O_4_.

## 2. Materials and Methods

### 2.1. Materials

Medical Ti foils (0.1 mm thickness, 10 mm in length and width, 99.9%) were purchased from Haiyuan Research Metals (Dongwan, China). Chloride hexahydrate (CoCl_2_·6H_2_O, 99.0%), potassium nitrate (KNO_3_, 99.0%), ammonium chloride (NH_4_Cl, 99.5%), sodium phosphate monobasic dihydrate (NaH_2_PO_4_·2H_2_O, 99.0%), ethanol absolute (CH_3_CH_2_OH, 99.7%), and copper chloride (CuCl_2_·2H_2_O, 99.0%) were bought from Chemical Reagent Co., Ltd. (Tianjin, China). Dopamine hydrochloride (C_8_H_11_NO_2_·HCl, 98.0%) and dimethyl sulfoxide (DMSO, 99.7%) were acquired from Macklin Biochemical Co., Ltd. (Shanghai, China). Tris base (C_4_H_11_NO_3_, 99.9%) was obtained from Gentihold Biotechnology Co., Ltd. (Beijing, China). In addition, a phosphate buffer solution (PBS) was purchased from Bio-Channel Biotechnology Co., Ltd. (Nanjing, China). Nutrient broth (NB) and nutrient agar (NA) were purchased from Hope Bio-Technology Co., Ltd. (Qingdao, China). Alternatively, the as-prepared NA plates could be stored at 4 °C for further use.

### 2.2. Characterization

The scanning electron microscope (SEM) with Quanta 200FEG (Thermo Fisher Scientific Inc., Waltham, MA, USA) and field emission transmission electron microscope (FETEM) with JEM-2100F (JEOL, Tokyo, Japan) were employed to analyze the surface morphology and compositions in the as-prepared samples, respectively. The high voltage was ranging from 10 to 20 kV. And the working distance was between 10 and 20 mm. The physical phases were recognized by X-ray diffraction (XRD, X’Pert PRO, Almelo, The Netherlands) with slow scanning modes at 3°/min in the range of 10 to 90°. The chemical element components were detected by X-ray photoelectron spectroscopy (XPS, ESCALAB 250Xi, Thermo Fisher Scientific Inc., Waltham, MA, USA). The electron paramagnetic resonance spectra (EPR) were conducted on a Bruker A300 spectrometer (A300-10/12, Ettlingen, Germany) with the X-band at a frequency of 9.853 GHz. The Raman spectra were acquired by a confocal Raman spectrometer (inVia-Reflex, New Mills, UK) with excitation wavelengths of 532, 633, or 785 nm. The chemical structure was determined with a Fourier transform infrared spectrometer (FTIR, Nicolet is50, Green Bay, WI, USA) using KBr blanks as controls in the range of 4000 to 400 cm^−1^. The optical properties of the samples were measured with an ultraviolet-visible-near-infrared (UV-Vis-NIR) spectrophotometer (U-4100, Tokyo, Japan) at a scanning rate of 600 nm/min. The fluorescence spectra were performed on a fluorescence spectrometer (Fluoromax-4, Irvine, CA, USA) with an excitation wavelength at 315 nm. The zeta potential was measured by Zetasizer Nano-ZS90 (Malvern Instruments, Malvern, UK). An atomic force microscope (AFM) was used on Agilent 5500 (Santa Clara, CA, USA). The topography images were used to measure the root mean square roughness (RMS) by Gwyddion 2.65 software.

### 2.3. Synthesis of Co_3_O_4_ Nanosheets on Ti foils

Medical Ti foils (10 mm × 20 mm × 0.1 mm) were polished sequentially with silicon carbide (SiC) sandpaper (1000, 2000, and 3000#) and cleaned by ultrasonication with anhydrous ethanol and deionized water for 10 min, respectively. Cleaned Ti foils were oven-dried at 50 °C and stored at room temperature for further use. Typically, Co_3_O_4_ precursors were prepared by the electrochemical deposition method in a three-electrode system including Ti foil as the working electrode, Pt foil as the counter electrode, and Ag/AgCl as the reference electrode. Herein, the electrolyte solution contained CoCl_2_ (0.02 mol/L), KNO_3_ (0.04 mol/L), and NH_4_Cl (0.2 mol/L). Also, the chronopotentiometry method was used to deposit the Co_3_O_4_ precursors at a current of 12.5 mA. After deposition, the Co_3_O_4_-precursor-modified Ti foils were placed in a quartz tube furnace and then calcined at 450 °C for 2 h under an N_2_ atmosphere to obtain porous Co_3_O_4_ nanosheets.

### 2.4. Fabrication of O_v_-Co_3_O_4_ and Cu-O_v_-Co_3_O_4_ Nanocoatings

The porous Co_3_O_4_ nanosheets were immersed in a CuCl_2_ (4 mmol/L) aqueous solution at room temperature for 24 h, which were denoted as Cu-Co_3_O_4_. Subsequently, the Co_3_O_4_ and Cu-Co_3_O_4_ nanosheets were treated by H_2_ plasma (3 sccm) at 30 W for 10 min under a vacuum of 30 Pa (named as O_v_-Co_3_O_4_ and Cu-O_v_-Co_3_O_4_, respectively).

### 2.5. Preparation of PDA/Cu-O_v_-Co_3_O_4_ Nanocoatings

The as-prepared Cu-O_v_-Co_3_O_4_ nanosheets were immersed in a dopamine hydrochloride solution (0.75 mg/mL) containing 1.5 mg/mL Tris (pH 8.5) for 24 h to form PDA films, which were named as PDA/Cu-O_v_-Co_3_O_4_.

### 2.6. Photothermal Experiment

Different samples (10 mm × 10 mm) were placed into a 24-well plate with 500 μL of PBS to determine the photothermal property, and each well was irradiated by an 808 nm NIR laser (1.5 W/cm^2^, 10 min). Subsequently, the photostability curves were also conducted under the NIR irradiation (1.5 W/cm^2^, 10 min) with five cycles of a heating-cooling test. What’s more, photothermal tests with different power densities (0.5, 1.0, 1.5, and 2.0 W/cm^2^) were also documented separately. Notably, all the thermal mapping was monitored via a thermal imager (FLIR i7, Washington, DC, USA) together with a cell phone to steadily record the actual temperature change on surfaces every 30 s during the process.

### 2.7. Antibacterial Activity Assessment

*E*. *coli* (ATCC 25922, China) from Shanghai Bioresource Collection Center (SHBCC) (Shanghai, China) and *S*. *aureus* (CMCC(B) 26003, China) from Shanghai Yingxin laboratory equipment Co., Ltd. (Shanghai, China) were selected as assay strains whose antibacterial activity was validated using the plate-counting method. The two thawed strains were firstly incubated in a sterilized NB medium in a 37 °C shaker at 120 rpm and then gathered until the concentration of bacterial suspension reached ~10^8^ colony-forming units per milliliter (CFUs/mL). In the meantime, five groups of different types of samples were placed in a 24-well plate, respectively, and sterilized together via ultraviolet light for an hour. Afterward, 500 μL of an as-prepared bacterial solution was dropped onto the surface of each sample, respectively, and co-incubated in a 37 °C incubator for 6 h. To maintain humidity and prevent the evaporation of the solution, between the wells of placed samples were full of sterile deionized water. Then, the samples were handled with the NIR laser (808 nm, 1.5 W/cm^2^) for 10 min, while the thermal imager was exposed to recording the temperature difference.

Furthermore, each sample was transferred to the containers with 2 mL of PBS before ultrasonic detachment (150 W, 50 Hz), and adherent bacteria were released from substrates after 5 min. After sequential dilutions, 40 μL of the bacterial liquids was dropped onto standard NA plates and spread well, then incubated at 37 °C overnight. Ultimately, bacterial colonies on the plates were captured in photos and counted. The relevant antibacterial rate was calculated utilizing the following formula: Antibacterial rate $\left(R\right)=\left(C-E\right)/E\times 100\%$, where *C* is the colony amount of the control group (Ti), and *E* is the colony amount of the modified samples.

In order to study the morphology of the bacteria attached to the sample surface, the bacteria on samples were firstly fixed with a 4% paraformaldehyde (Biosharp, Beijing, China) solution at 4 °C for 4 h, and then gradually dehydrated utilizing a gradient ethanol solution (10, 30, 50, 70, 80, 90, and 100%) for 15 min. Finally, all samples were dried overnight and covered with gold for further SEM observation.

The bacteria live/dead staining was performed with SYTO9 Green Fluorescent Nucleic Acid Stain (Mao Kang, Shanghai, China) and Propidium Iodide (PI) Red Fluorescent Nucleic Acid Stain (Beyotime, Shanghai, China) to visualize the antibacterial ability on different samples. The specific methods for dye staining were in accordance with the instructions. After a series of operations, the live/dead bacteria were recorded utilizing a confocal laser scanning microscope (CLSM, Zeiss LSM880, Jena, Germany).

### 2.8. Analysis of Antibacterial Mechanism

#### 2.8.1. Bacterial Membrane Permeability Assay

To determine the integrity of the bacterial membrane, 8-anilino-1-naphthalenesulfonic acid (ANS, 96%, Aladdin, Shanghai, China) was devoted to evaluating the change in outer membrane permeability towards *E. coli*, due to the outer membrane unique to the Gram-negative bacteria. In contrast, o-Nitrophenyl β-D-galactopyranoside (ONPG, 98%, Yuanye, Shanghai, China) was employed to measure bacterial inner membrane change in the above two species of bacteria. Briefly, after the antibacterial process, each bacterial solution was disposed of a 500 μL ONPG solution (0.75 mol/L in NaH_2_PO_4_ buffer, pH 7.0). Finally, the yellow supernatant was removed and detected at the absorption of 420 nm (OD_420_) with a SPARK multifunctional microplate reader (Tecan, Austria GmbH, Kärnten, Austria).

#### 2.8.2. Bacterial Total ROS Level Detection

The generation of total ROS levels within the bacteria was determined by a 2′, 7′-dichlorodihydrofluorescein diacetate (H_2_DCFDA) probe and a ROS Assay Kit (Cat#BL714A, Biosharp, Beijing, China). After irradiation for 10 min, the bacterial suspension was co-cultured with H_2_DCFDA (10 μmol/L in PBS) at 37 °C for 30 min, standing in the dark. After that, 150 μL of bacterial solutions was seeded onto a 96-well plate in order and analyzed by a SPARK multifunctional microplate reader (Tecan, Austria GmbH, Kärnten, Austria) at the excitation/emission wavelength of 488/525 nm.

#### 2.8.3. Singlet Oxygen Measurement

The generation of singlet oxygen (^1^O_2_) from different sample surfaces was related to 1, 3-Diphenylisobenzofuran (DPBF). Firstly, 500 μL of DPBF (5 mmol/L in DMSO) was co-incubated with different samples in darkness for 10 min. After 808 nm of irradiation, the supernatant was collected and measured at the absorption of 420 nm (OD_420_) with a SPARK multifunctional microplate reader (Tecan, Austria GmbH, Kärnten, Austria).

#### 2.8.4. Hydroxyl Radical Evaluation

The production of the hydroxyl radical (•OH) was measured with disodium terephthalate (DST). Different samples were immersed with 5mL of DST (0.5 mmol/L in deionized water) for 10 min in advance. After, with/without 808 nm of irradiation, the ability to generate •OH was detected with the fluorescence spectrometer with the excitation/emission wavelength of 315 nm/425 nm.

#### 2.8.5. Bacterial Protein Leakage Assay

The protein assay kit (Cat#PC0020, Solarbio, Beijing, China) based on the bicinchoninic acid (BCA) method was carried out to study the bacterial protein leakage after irradiation, which was detected with absorbance values at 562 nm (OD_562_) by a SPARK multifunctional microplate reader (Tecan, Austria GmbH). In brief, after being irradiated for 10 min, respectively, 20 μL of as-treated bacterial solutions was removed and added into a 48-well plate with 200 μL of a solution, placing it in a 37 °C incubator for 30 min. Finally, withdraw the supernatant immediately and analyze the corresponding bacterial protein leakage by a SPARK multifunctional microplate reader (Tecan, Austria GmbH, Kärnten, Austria) at 562 nm.

#### 2.8.6. Bacterial ATP Level Test

The enhanced ATP assay kit (Cat#S0026, Beyotime, Shanghai, China) was applied to research the bacterial metabolic activity. After irradiation, the already treated bacteria solution was collected and co-cultured with 100 μL of the lysate before being sonicated for 3 min to fully lyse. Lastly, suck out 20 μL of the supernatant and 100 μL of the working solution, inject them into a 96-well black plate together, and then measure the ATP levels through the program “chemiluminescence” via a SPARK multifunctional microplate reader (Tecan, Austria GmbH, Kärnten, Austria).

### 2.9. Metal Ion Release Test

The metal ion release behavior was determined by inductively coupled plasma optical emission spectrometry (ICP-OES, iCAP 7400, Thermo Fisher Scientific Inc., Waltham, MA, USA). The sample (10 mm × 10 mm) was placed into centrifuge tubes containing 10 mL of PBS (pH 7.4) and simultaneously immersed in a 37 °C water bath for 3, 6, 9, and 12 h, where the untreated PBS was used as a control group. At each time, the sample was removed and the corresponding solutions were transferred to a −80 °C refrigerator for freezing. After that, all sample tubes were freeze-dried for 24 h. Finally, 10 mL of HNO_3_ (2%) was added to each tube and diluted 3-fold for further ICP detection.

### 2.10. Cytotoxicity Evaluation

Mouse fibroblasts (L-929, Beijing, China) were used to study the biocompatibility of different samples [21]. The L-929 cells were cultured in a 37 °C incubator with 5% CO_2_ for 24 h and the cell medium was changed every other day. Subsequently, the cytotoxicity towards L-929 fibroblasts was evaluated by methyl thiazolyl tetrazolium (MTT) assays.

Briefly, 500 μL (3.0 × 10^4^ cells/mL) of the L-929 suspension was added onto the surface of various sterilized samples in a 24-well plate, respectively, and co-incubated for 1, 2, and 3 days, ensuring the sample surface was completely immersed by the cell medium. At each time point, the cell medium was removed before 50 μL of MTT (0.5 mg/mL) was added into the well and returned to the incubator for 4 h in darkness. The supernatant was carefully sucked out twice and then dissolved in 150 μL of DMSO, also being shaken, and vibrated for another 15 min. Finally, the supernatant was evaluated at the absorption of 490 nm (OD_490_) on a SPARK multifunctional microplate reader (Tecan, Austria GmbH, Kärnten, Austria) while the cell medium was applied as the control.

## 3. Results and Discussions

### 3.1. Synthesis and Physicochemical Properties

**Figure 1:**
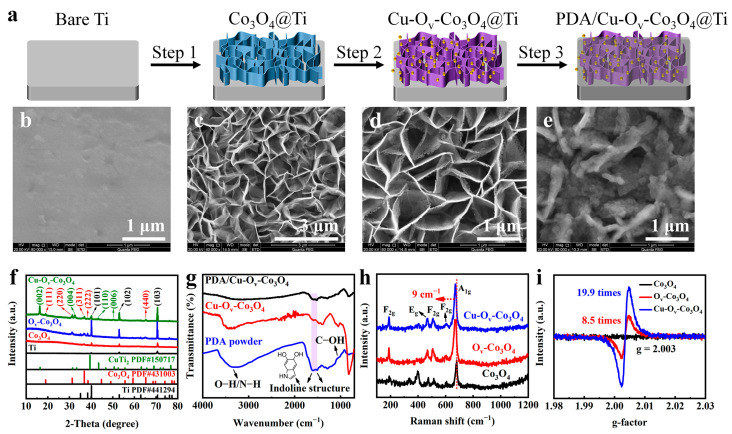
Characterization of PDA/Cu-O_v_-Co_3_O_4_@Ti. (**a**) Schematic procedure of antibacterial nanocoating. SEM images of (**b**) bare Ti, (**c**) Co_3_O_4_@Ti, (**d**) Cu-O_v_-Co_3_O_4_@Ti, and (**e**) PDA/Cu-O_v_-Co_3_O_4_@Ti. (**f**) Corresponding XRD patterns, (**g**) FTIR spectra, (**h**) Raman spectra, and (**i**) EPR spectra.

As illustrated in Figure 1a, the electrodeposition and H_2_ plasma annealing process were applied to synthesize Co_3_O_4_ ultrathin nanosheets on as-polished Ti foils’ surface. After modifying with PDA in Figure S1a–e, it could be found that the color on the PDA/Cu-O_v_-Co_3_O_4_ surface obviously varied from brown to black. The morphology and structure features on different samples were observed by the SEM. As demonstrated in Figure 1b and Figure S1f, the surface of bare Ti foils was smooth. In contrast, after electrodeposition (Step 1 in Figure 1a), Co_3_O_4_ (Figure S1g and Figure 1c) ultrathin nanosheets were successfully prepared on the Ti surface and its morphology (Figure S1h and Figure 1d) did not change after calcination treatment, with an average pore size of approximately 1.5 μm. Interestingly, the surface of the O_v_-Co_3_O_4_ (Figure S1h) and Cu-O_v_-Co_3_O_4_ sample (Figure S1i and Figure 1d) displayed nanopores (Step 2 in Figure 1a), which were caused by high-energy H_2_ plasma. It is assumed that Cu doping and plasma treatment were able to regulate O_v_ concentration on the Co_3_O_4_ surface. Engineering of constructive surface defects allows us to optimize the energy band structure productively, thereby enhancing the optical absorption capacity and the photothermal conversion efficiency [22,23]. SEM images in Figure S1j and Figure 1e showed that a layer of polymer nanofilm was covered on the surface of nanosheets (Step 3 in Figure 1a), resulting from the polymerization of dopamine.

XRD was used to confirm the phase patterns related to as-prepared nanosheets. As revealed in Figure 1f, diffraction peaks at 40.17, 53.00, and 70.66° corresponded to (101), (102), and (103) crystal facets, which were matched with Ti (PDF#44-1294). The diffraction peaks for Co_3_O_4_@Ti and O_v_-Co_3_O_4_@Ti were observed at 19.00, 31.27, 36.85, 38.53, and 65.25°, which were associated with the (111), (220), (311), (222), and (440) facets of Co_3_O_4_ (PDF#43-1003) [24,25]. In addition, for Cu-doped O_v_-Co_3_O_4_ (named as Cu-O_v_-Co_3_O_4_), the (002), (004), (110), and (006) peaks were found, in which the presence of Co_3_O_4_ and CuTi_2_ were observed assuming that Cu doping destroyed the long-range order of O_v_-Co_3_O_4_. The mixture was mainly caused by high-energy H_2_ plasma treatment.

FTIR and Raman spectra were used to verify the presence of PDA. As presented in Figure 1g, the three bands at 1543, 1616, and 3315 cm^−1^ were related to the vibration mode of C−O, C=O, and O−H/N−H groups in PDA, respectively [26,27]. In contrast, no other bands for PDA were found in FTIR spectra of Cu-O_v_-Co_3_O_4_@Ti, indicating the successful preparation of PDA coating. Furthermore, Raman spectra were carried out to compare various samples before and after H_2_ plasma treatment and Cu doping. As seen in Figure S2, two bands from 456 to 756 cm^−1^ appeared compared with PDA powder besides the stretching and deformation of PDA in the range of 1350 to 1650 cm^−1^ [4,10]. These emerging bands at 193, 467, 511, 606, and 678 cm^−1^ were ascribed to F^1^_2g_, E_2g_, F^2^_2g_, F^3^_2g_, and A_1g_ symmetry modes of Co_3_O_4_, respectively [28,29]. It is worth noting that A_1g_ modes in O_v_-Co_3_O_4_ and Cu-O_v_-Co_3_O_4_ were red-shifted by 9 cm^−1^, probably owing to the introduction of O_v_.

To verify the existence of O_v_, EPR was conducted to explore the O_v_ quantity contained in Figure 1i as it is well known that a stronger signal represents more O_v_ sites. The EPR signal at g = 2.003 was caught in both O_v_-Co_3_O_4_@Ti and Cu-O_v_-Co_3_O_4_@Ti, the intensity of which was around 8.5 and 19.9 times that of Co_3_O_4_@Ti, respectively. Such improvements further proved that massive O_v_ was successfully introduced in the O_v_-Co_3_O_4_ sample, which was further increased in Cu-O_v_-Co_3_O_4_ due to Cu doping and H_2_ plasma treatment. O_v_ had been demonstrated to substantially decelerate the photogenerated carriers’ recombination rates, which resulted in the generation of more ROS, and then the photocatalytic antibacterial performance would be enhanced [22,30,31].

**Figure 2:**
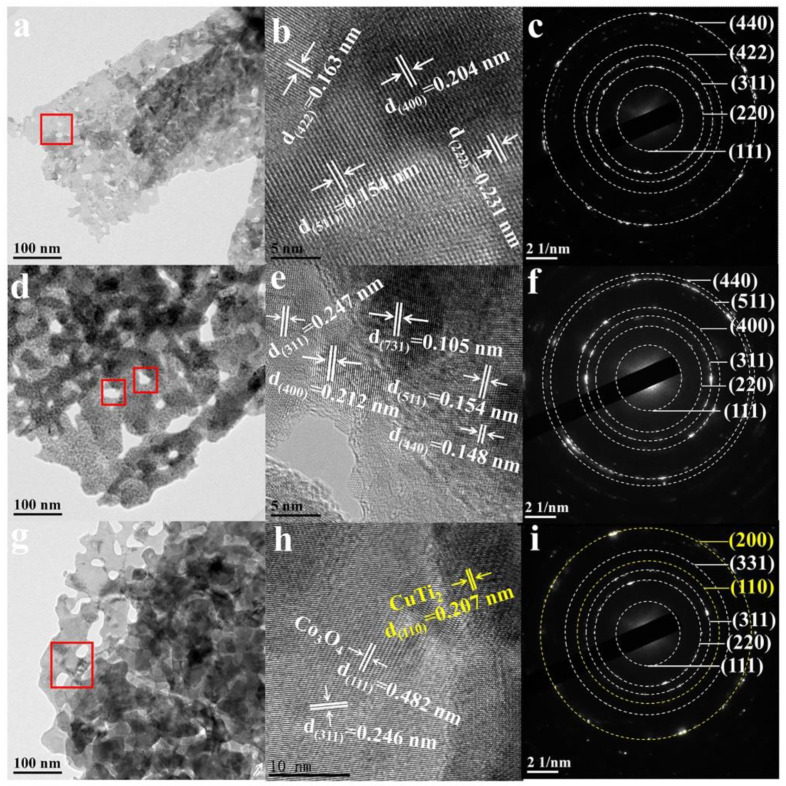
TEM characterization of the synthesized nanosheets. (**a**,**d**,**g**) TEM, (**b**,**e**,**h**) HRTEM, and (**c**,**f**,**i**) SAED images of Co_3_O_4_@Ti, O_v_-Co_3_O_4_@Ti, and Cu-O_v_-Co_3_O_4_@Ti, respectively. Red squares represent nanopores.

A TEM was further used to investigate the morphology and crystalline boundary. As seen in Figure 2a,d,g, the introduction of O_v_ led to a large amount of nanopores on nanosheets, as indicated by red squares. These pores would favor the exposure of active sites for the generation of ROS. HRTEM in Figure 2b,e shows the crystalline facets of (222), (400), (422), (511), (311), and (440), ascribed to Co_3_O_4_ [8]. It is seen that the main crystalline phase in O_v_-Co_3_O_4_@Ti was still Co_3_O_4_ in Figure 2f. Besides the crystalline facets of Co_3_O_4_, the interlayer distance of 0.207 nm was found in Cu-O_v_-Co_3_O_4_@Ti in Figure 2h, corresponding to the crystalline CuTi_2_ (110) facet. Eventually, SAED patterns in Figure 2i disclosed the coexistence of Co_3_O_4_ and CuTi_2_, consistent with XRD results.

**Figure 3:**
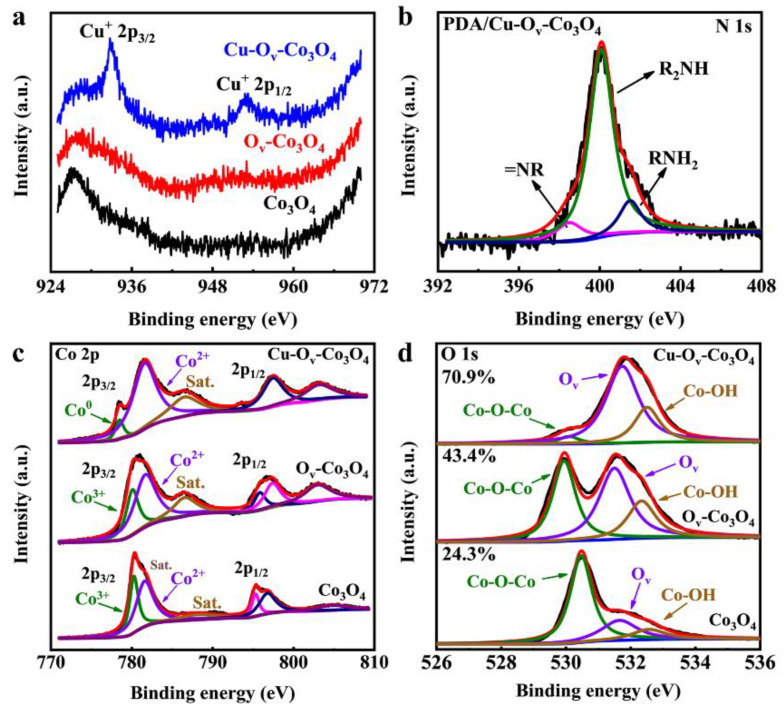
High-resolution XPS spectra for (**a**) Cu 2p, (**b**) N 1s, (**c**) Co 2p, and (**d**) O 1s. Different color lines represent elements with different valence states.

The XPS analysis was carried out to evaluate the chemical component and changes in element status on the sample surface. As observed in Figure S3, XPS spectra revealed that the PDA/Cu-O_v_-Co_3_O_4_ sample consisted of C, O, Co, N, and Cu elements. The fine deconvolution of Cu 2p in Figure 3a shows two peaks at a binding energy of 932.74 and 952.68 eV, respectively, consistent with the spin-orbital doublet peaks of Cu^+^ 2p_3/2_ and Cu^+^ 2p_1/2_, indicating that Cu^+^ ions were generated during H_2_ plasma treatment [32,33]. N 1s XPS spectra in Figure 3b display three peaks at 398.61, 400.14, and 401.53 eV, respectively, belonging to tertiary/aromatic (=N−R), secondary (R−NH−R), and primary (R−NH_2_) amine, respectively, further determining the successful attachment of PDA [34]. Co 2p XPS spectra of Co_3_O_4_ and O_v_-Co_3_O_4_ in Figure 3c were deconvoluted into four peaks, indicating the coexistence of Co^2+^ and Co^3+^ on the sample surface [8,35,36]. Interestingly, Co^0^ was found in Cu-O_v_-Co_3_O_4_, which might be caused by the reduction in high-energy H_2_ plasma. O 1s XPS spectra in Figure 3d indicate that the peaks for the Co−O−Co bond in Cu-O_v_-Co_3_O_4_@Ti decreased greatly compared with Co_3_O_4_@Ti and O_v_-Co_3_O_4_@Ti [7,9,36]. In addition, the peaks related to O_v_ increased from 24.3 to 43.4% after introducing O_v_ by H_2_ plasma, and finally increased to 70.9%, demonstrating that a large amount of O_v_ sites were generated in Cu-O_v_-Co_3_O_4_@Ti.

Moreover, water contact angle measurement was conducted to verify the wettability of different samples in Figure S4. The Ti substrate exhibited hydrophilicity and the contact angle was 73.73°. However, Co_3_O_4_ and O_v_-Co_3_O_4_ nanosheets became hydrophobic (149.44 and 153.78°), which might be caused by the nanostructured surface [37,38]. After the introduction of Cu, the contact angle decreased to 79.96° in the Cu-O_v_-Co_3_O_4_@Ti, and that of PDA/Cu-O_v_-Co_3_O_4_@Ti further decreased to 34.41° after PDA coating. It was assumed that the hydrophilic surface of PDA/Cu-O_v_-Co_3_O_4_ could facilitate the infiltration of the electrolyte solution and subsequent attachment of bacteria, then triggering the next sterilization.

### 3.2. Photothermal Performance

**Figure 4:**
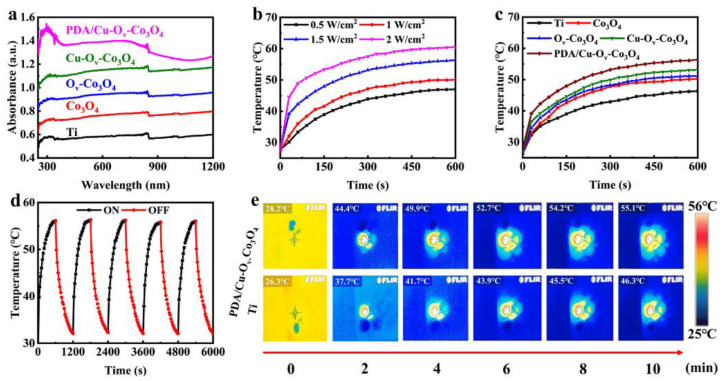
Evaluation of photothermal properties. (**a**) Ultraviolet–visible–Diffuse-Reflectance Spectra (UV-Vis-DRS) of all samples. (**b**) The heating curves of PDA/Cu-O_v_-Co_3_O_4_@Ti treated with different power densities (0.5, 1.0, 1.5, and 2.0 W/cm^2^) for 10 min (808 nm, 1.5 W/cm^2^). (**c**) The heating curves of different samples upon NIR irradiation for 10 min (808 nm, 1.5 W/cm^2^). (**d**) The five cycle curves irradiated with NIR irradiation (808 nm, 1.5 W/cm^2^). (**e**) Real-time thermal mapping corresponding to bare Ti foils and PDA/Cu-O_v_-Co_3_O_4_@Ti.

The NIR absorption properties were investigated by the UV-Vis-DRS in Figure 4a. The absorbance intensity at 808 nm increased by 11.5, 24.4, and 48.3%, respectively, after the modification of Co_3_O_4_, O_v_-Co_3_O_4_, and Cu-O_v_-Co_3_O_4_ on Ti, which was caused by the decreased band gap due to the presence of O_v_. According to solid physics, O_v_ could generate defective energy around the Fermi level, thus resulting in a smaller band gap [31,39]. The absorbance of PDA/Cu-O_v_-Co_3_O_4_ further increased by 77.0% compared to bare Ti foils after coating a thin layer of PDA nanofilm. When irradiated by an 808 nm laser in Figure 4b, the surface temperature for PDA/Cu-O_v_-Co_3_O_4_ in PBS was dependent on laser power densities of 0.5, 1.0, 1.5, and 2.0 W/cm^2^, respectively. To protect normal cell tissue, all thermal tests were performed at 1.5 W/cm^2^ for 10 min in Figure 4c. In the case of PDA/Cu-O_v_-Co_3_O_4_@Ti, the temperature finally increased to 55.1 °C within 10 min, higher than that of control groups consisting of Ti (46.3 °C), Co_3_O_4_@Ti (50.2 °C), O_v_-Co_3_O_4_@Ti (51.2 °C), and Cu-O_v_-Co_3_O_4_@Ti (53.1 °C), respectively. The thermal stability was measured in Figure 4d. The highest temperature at the fifth cycle was almost unchanged compared to that in the first cycle, indicating good thermal stability in the aqueous solution. The real-time thermal mapping in Figure 4e reflects the photothermal region of PDA/Cu-O_v_-Co_3_O_4_@Ti and bare Ti foils, and the real-time thermal mapping of the other three samples is shown in Figure S5. It is seen that the surface temperature was even for all cases. However, the PDA/Cu-O_v_-Co_3_O_4_@Ti substrate clearly exhibited higher temperature than the medical bare Ti foils.

### 3.3. Antibacterial Activity

Next, the antibacterial performance was evaluated using different substrates. Based on the above characterization, the bacteria were synergistically sterilized by the photothermal effect, Fenton-like activity of Cu^+^ ions, and photodynamic effect of the semiconductor. The standard plate-counting experiment was conducted to study the antibacterial efficacy. In our study, *E. coli* (Gram-negative) and *S. aureus* (Gram-positive) were utilized as the strain types of investigation. Firstly, the dynamic growth curves of *E. coli* and *S. aureus* were measured on a 24-well plate by optical density at 600 nm (OD_600_) methods and the results are displayed in Figure S6, and bacteria in the logarithmic phase of microbial growth were selected for the following study.

**Figure 5:**
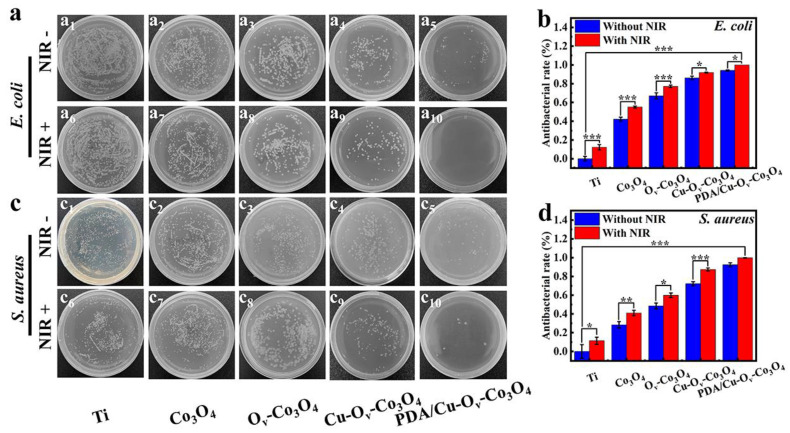
Antibacterial activity. Spread plate images against (**a**) *E. coli* and (**c**) *S. aureus*. The corresponding antibacterial rates against (**b**) *E. coli* and (**d**) *S. aureus*. *, ** and *** represent *p* < 0.05, *p* < 0.01 and *p* < 0.001, respectively.

After co-incubation with various samples and with or without 808 nm of irradiation, the amounts of *E. coli* and *S. aureus* on agar plates are shown in Figure 5a,c. The antibacterial efficacy results are shown in Figure 5b,d. In the dark condition, the Co_3_O_4_ nanosheets expressed weak toxicity against *E. coli* (44.22%) and *S. aureus* (22.27%) in comparison with bare Ti foils, whereas after the introduction of O_v_, the antibacterial capability had an increase on *E. coli* (67.05%) and *S. aureus* (47.27%), respectively. In addition, the inhibition rate towards *E. coli* and *S. aureus* for Cu-O_v_-Co_3_O_4_@Ti increased to around 86.14 and 72.69%, and further increased to 94.17 and 94.93% for PDA/Cu-O_v_-Co_3_O_4_@Ti. Under NIR (808 nm, 1.5 W/cm^2^) conditions, the antibacterial activity for bare Ti foils was improved against *E. coli* (12.28%) and *S. aureus* (11.85%), whereas the inhibition rate for Co_3_O_4_@Ti increased to 55.14 and 37.58%, respectively. Similarly, the O_v_-Co_3_O_4_ sample showed a higher inhibition rate to *E. coli* (77.08%) and *S. aureus* (53.43%) as well. Moreover, after the introduction of Cu, the Cu-O_v_-Co_3_O_4_ sample killed 93.27% of *E. coli* and 91.85% of *S. aureus*. Most importantly, the PDA/Cu-O_v_-Co_3_O_4_ sample demonstrated the highest inhibition rate of ca. 100% against both bacteria. Compared to the reported Ti-PDA/BP/ZnO, TiO_2_/MoS_2_/PDA/RGD, CpTi-SiO_2_-3Cu, and Ti-Co_15_ coatings, the good antibacterial result of PDA/Cu-O_v_-Co_3_O_4_ was speculated to be attributed to the ROS, localized hyperthermia, and metal ion release effects [8,40,41,42].

Basically, in order to kill *E. coli*, the direct heating temperature should not be lower than 50 °C; otherwise, there is no substantial threat to the viability of *E. coli*. On the other hand, only at an irradiation temperature of up to 88.8 °C and a duration of 15 min for the NIR laser could *S. aureus* be completely killed [43]. However, higher localized temperature will cause serious damage to normal cells. Thus, as stated above, we would like to kill bacteria by utilizing a synergistic strategy at relatively lower temperature in our study.

**Figure 6:**
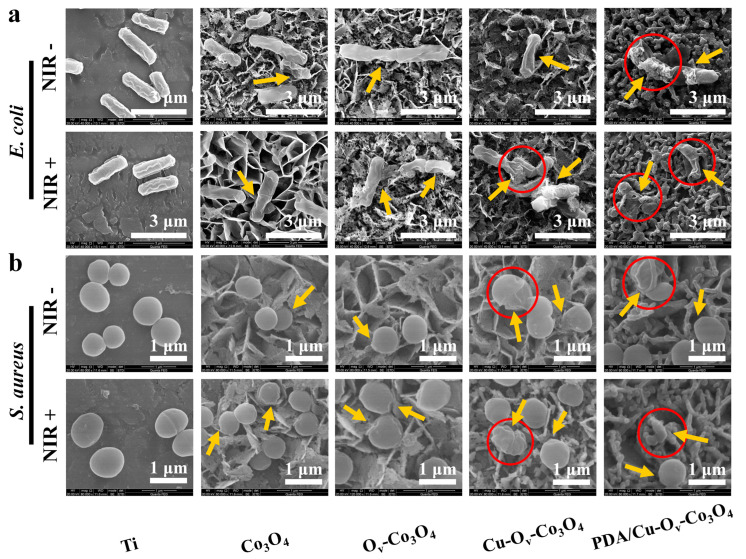
Bacterial morphology visualization. SEM images of (**a**) *E. coli* and (**b**) *S. aureus* with and without NIR irradiation by various substrates. Red circles indicated bacterial deformation or rupture.

The SEM was capitalized on to observe two bacterial morphologies and integrities on the sample surface. As seen in Figure 6a,b, the bacteria were detected on bare Ti foils after co-culturing for 6 h, exhibiting smooth and complete rod-shaped *E. coli* and spherical-shaped *S. aureus* without or with NIR laser irradiation. However, a slightly wrinkled and distorted structure change was observed in the Co_3_O_4_-based group (yellow arrows), respectively, ascribed to its physical interaction between bacterial membranes and nanosheets. In sharp contrast, under irradiation, Co_3_O_4_+NIR, O_v_-Co_3_O_4_+NIR, Cu-O_v_-Co_3_O_4_+NIR, and PDA/Cu-O_v_-Co_3_O_4_+NIR groups caused the deformation of the bacterial membrane. It was noticed that the most severe disruptive effects containing lesions, holes, and shrinkage were observed in the PDA/Cu-O_v_-Co_3_O_4_ group, which were related to its multifunctional antibacterial activity.

The antibacterial effect was further evaluated through a live/dead fluorescence staining assay. The live bacteria could be stained to green by STYO 9, and the dead bacteria showed a red color after PI staining. When both staining reagents are present, the fluorescence intensity originating from PI will be more intense. Figure S7 shows CLSM images on the sample surface after co-culturing for 6 h. It is shown that *E. coli* and *S. aureus* on the surface of Ti foils were stained with green with or without NIR irradiation. As for PDA/Cu-O_v_-Co_3_O_4_@Ti, after exposure to NIR irradiation, dead bacteria with red fluorescence were seen, indicating that *E. coli* and *S. aureus* were substantially killed.

**Figure 7:**
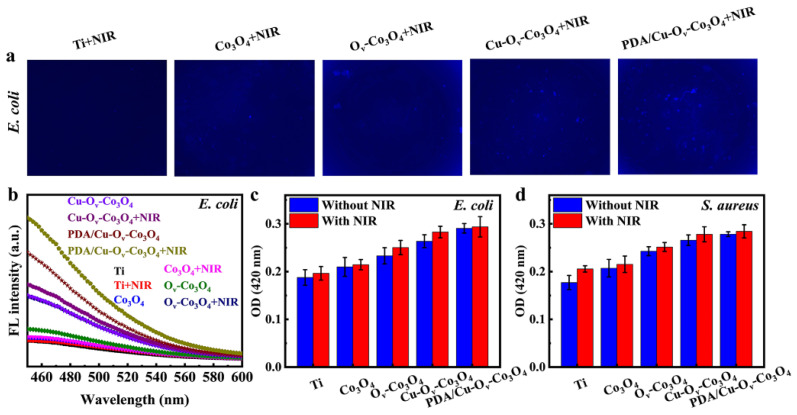
Evaluation of bacterial membrane permeability. (**a**) Fluorescence images of substrates covered with *E. coli*, which were tested by ANS probe with excitation length of 380 nm. (**b**) Corresponding fluorescence intensities. Bacterial inner membrane permeability of (**c**) *E. coli* and (**d**) *S. aureus* monitored by ONPG assay.

In addition, the bacterial membrane permeability was verified by ANS and ONPG analyses, respectively. ANS was utilized to determine the outer membrane permeability towards *E. coli*. Due to the difference in bacteria structure, the outer membrane was solely present in Gram-negative bacteria [44]. ANS was able to reinforce the fluorescence imaging via integrating with the hydrophobic district of the external membrane [45,46]. As displayed in Figure 7a, after being co-cultured with ANS, the Ti+NIR group was non-fluorescent, whereas blue fluorescence could be detected in the other four photothermal groups. In particular, as shown in Figure 7b, the PDA/Cu-O_v_-Co_3_O_4_+NIR group exhibited higher fluorescence intensity than the Ti+NIR group, Co_3_O_4_+NIR group, O_v_-Co_3_O_4_+NIR group, and Cu-O_v_-Co_3_O_4_+NIR group. It is assumed that the photothermal effect played a significant role in severely disrupting the outer membrane structure of *E. coli*, thus accelerating the bacterial death.

The changing permeability of the bacterial internal membrane was also evaluated by ONPG hydrolysis assays. The ONPG could be hydrolyzed by intracellular β-galactosidase (β-Gal) to produce galactose and yellow ο-nitrophenol (ONP) detected with a strong absorption peak at 420 nm [47,48,49]. As indicated in Figure 7c,d, the OD_420_ values for bare Ti were lower than 0.2 for both *E. coli* and *S. aureus*. However, the OD_420_ value for PDA/Cu-O_v_-Co_3_O_4_ increased by 55 and 57% for both *E. coli* and *S. aureus* compared with bare Ti in the dark, indicating enhanced inner membrane permeability. And upon NIR irradiation, the OD_420_ value further increased in both cases. The above results illustrated that the physical impedance of nanosheets and the photothermal effect synergistically enhanced the bacterial membrane permeability. Previous work also found that high temperature (around 50 °C) was able to enhance membrane permeability in the bacteria [40]. As a result, more external toxic substances such as ROS or metal ions could enter the cell interior and lead to its death [50].

**Figure 8:**
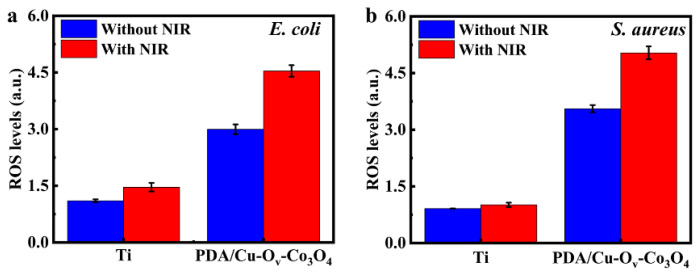
The relative intracellular ROS levels of (**a**) *E. coli* and (**b**) *S. aureus*.

Besides the enhanced membrane permeability, we next investigated the ROS levels in bacteria. After NIR irradiation, the ROS levels were detected by the H_2_DCFDA analysis. ROS in bacteria will oxidize DCFH to produce the fluorescein, named 2′, 7′-dichlorofluorescein (DCF), and the corresponding fluorescence intensity directly reflecting the intracellular ROS levels within the bacteria [13,51]. Figure 8a,b show that PDA/Cu-O_v_-Co_3_O_4_@Ti promoted the generation of DCF in the dark compared to that of bare Ti, and the intensity increased by 51.5% for *E. coli* and 41.5% for *S. aureus* upon NIR irradiation. This result indicated that bacteria underwent severe oxidative stress on the PDA/Cu-O_v_-Co_3_O_4_ substrate, thus inducing bacteria to produce more ROS. Hence, the antibacterial effect was partially attributed to internal damage induced by a considerable ROS attack within the bacteria, thus resulting in their oxidative stress.

Moreover, it was found in Figure S8 that the zeta potentials of pure *E. coli* and *S. aureus* suspensions were −34.46 and −27.56 mV. The zeta potentials of Co_3_O_4_@Ti, O_v_-Co_3_O_4_@Ti, Cu-O_v_-Co_3_O_4_@Ti, and PDA/Cu-O_v_-Co_3_O_4_@Ti were −16.60, −8.84, −6.38, and −10.97 mV, respectively. It is seen that the introduction of O_v_ and Cu increased the zeta potentials. PDA coating slightly decreased the zeta potential, which was attributed to the negatively charged •OH groups in PDA. In brief, the electrostatic repulsion between bacteria and PDA/Cu-O_v_-Co_3_O_4_@Ti was reduced compared with Co_3_O_4_@Ti, being conducive to capturing the bacteria [52].

**Figure 9:**
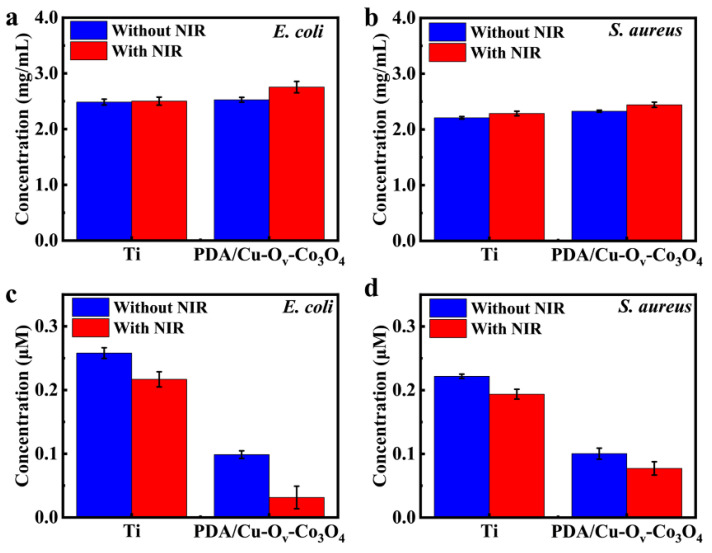
Protein levels released by (**a**) *E. coli* and (**b**) *S. aureus* and reduced ATP levels in (**c**) *E. coli* and (**d**) *S. aureus*.

As stated above, cell permeability was improved, which might lead to the protein leakage. Thus, the protein leakage assay was used to evaluate the protein levels in bacterial solutions. According to standard curves in Figure S9, the protein concentration was related to OD_562_ values. In general, higher OD_562_ values represent more protein released, which indicates that the severe disruption of the bacterial membrane or increase in membrane permeability to some extent occurred in the presence of antibacterial substrates. As shown in Figure 9a,b, PDA/Cu-O_v_-Co_3_O_4_@Ti after irradiation exhibited the highest protein leakage for both *E. coli* and *S. aureus*, suggesting that the bacterial cell membrane was severely damaged by the photothermal effect [53,54]. As a result, more protein leakage led to the metabolic imbalance and ultimate death of bacteria.

The presence of hyperthermia, ROS, and metal ions resulted in the imbalanced bacterial metabolism; thus, ATP levels might be downregulated. It could be said that the inactivation of bacteria cells was associated with the ATP deficiency [55,56]. Figure 9c,d display that a significant decrease in ATP concentration was observed for PDA/Cu-O_v_-Co_3_O_4_@Ti in the dark compared with bare Ti, and it kept decreasing under NIR irradiation. The downregulated ATP was sufficient to provide essential energy for bacterial proliferation during the cellular respiration and metabolism. Thus, the bacteria-respiratory process was severely disturbed upon NIR irradiation, thus leading to cell death.

**Figure 10:**
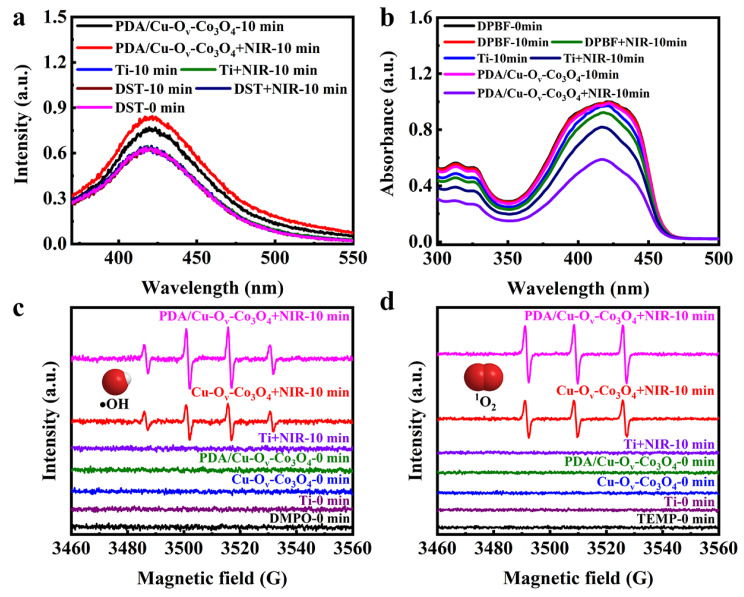
Photodynamic assessments. Identification of (**a**) •OH by fluorescence probe of DST and (**b**) ^1^O_2_ by DPBF. EPR tests of different samples trapped with (**c**) DMPO for •OH and (**d**) TEMP for ^1^O_2_, respectively (laser power density of 1.5 W/cm^2^, irradiation distance of 10 cm, and irradiation time of 10 min).

ROS was of great importance to cell death. Thus, the identification of various ROS was conducive to disclosing the antibacterial mechanism. The DST fluorescent probe was used as the trap to detect the production capability of •OH [57,58]. As shown in Figure 10a, the characteristic fluorescence peak of DST alone was negligible in the dark, indicating that DST itself did not generate •OH. In the case of bare Ti foils, the fluorescence intensity was almost unchanged. However, the fluorescence intensity for the PDA/Cu-O_v_-Co_3_O_4_ sample increased by 21.38% after incubating for 10 min in the dark, which was related to the self-supplied H_2_O_2_ due to the oxidation of PDA in the presence of solubilized O_2_. And this intensity further increased by 35.44% upon NIR irradiation, indicating that the photothermal effect could promote the generation of •OH. Cu-doped O_v_-Co_3_O_4_ as a photosensitizer might possess a photodynamic effect during irradiation. ^1^O_2_ was measured by monitoring the DPBF absorbance at 420 nm [59,60]. As seen in Figure 10b, the fluorescence intensity of pure DPBF and in the presence of bare Ti foils was almost unchanged when placed in the dark for 10 min. However, an obvious intensity decrease was observed when PDA/Cu-O_v_-Co_3_O_4_@Ti was irradiated by NIR irradiation for 10 min. It is assumed that an energy transfer occurred from O_2_ to ^1^O_2_ during NIR irradiation in semiconductive Cu-O_v_-Co_3_O_4_.

EPR was applied to solidly validate the presence of •OH and ^1^O_2_. Under dark conditions, neither the Cu-O_v_-Co_3_O_4_ nor PDA/Cu-O_v_-Co_3_O_4_ sample could present any EPR signals corresponding to •OH or ^1^O_2_. However, the EPR signals of the Cu-O_v_-Co_3_O_4_@Ti sample were significantly enhanced upon NIR irradiation. As seen in Figure 10c, the intensities show 1:2:2:1, which was ascribed to the typical •OH peaks. The 1:1:1 profile in Figure 10d means that the presence of ^1^O_2_ was due to photodynamic effects. Importantly, both signal intensities obviously increased towards PDA/Cu-O_v_-Co_3_O_4_@Ti after irradiation for 10 min, indicating the generation of larger amounts of either •OH or ^1^O_2_ than that of the Cu-O_v_-Co_3_O_4_@Ti sample.

### 3.4. Cell Compatibility

**Figure 11:**
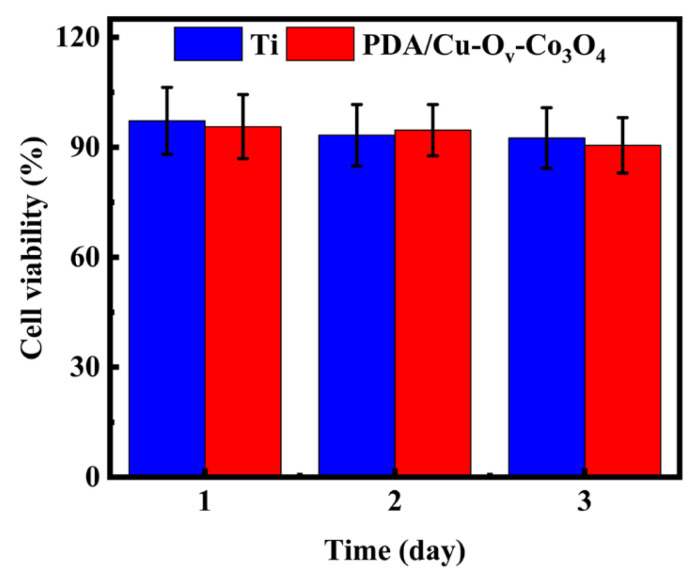
Cell viability of L-929 on bare Ti and PDA/Cu-O_v_-Co_3_O_4_ samples.

To further support the future application, the cytotoxicity of bare Ti foils and PDA/Cu-O_v_-Co_3_O_4_@Ti was measured towards L-929 cells by MTT assays for 3 days. In Figure 11, the cell viability of the Ti group is about 92.5% at 3 days, indicating its negligible cytotoxicity towards L-929 cells. In the whole culturing process, the cell viability of the PDA/Cu-O_v_-Co_3_O_4_@Ti sample was about 90.5% after culturing for 3 days. Consequently, PDA/Cu-O_v_-Co_3_O_4_@Ti could be favorable to the normal cell propagation and growth, showing higher biocompatibility. In addition, Co and Cu ions were slowly released into the PBS over 12 h and the concentrations were determined by ICP tests. As seen in Figure S10, the concentrations of Co and Cu ions for PDA/Cu-O_v_-Co_3_O_4_@Ti were 0.201 and 0.179 μg/mL after 6 h, followed by 0.533 and 0.290 μg/mL after 12 h, respectively. Combined with the spread plate results, it was demonstrated that the released metal ions in solutions might trigger the bacterial cytotoxicity along with the contact sterilization.

### 3.5. Synergistic Antibacterial Mechanism

**Figure 12:**
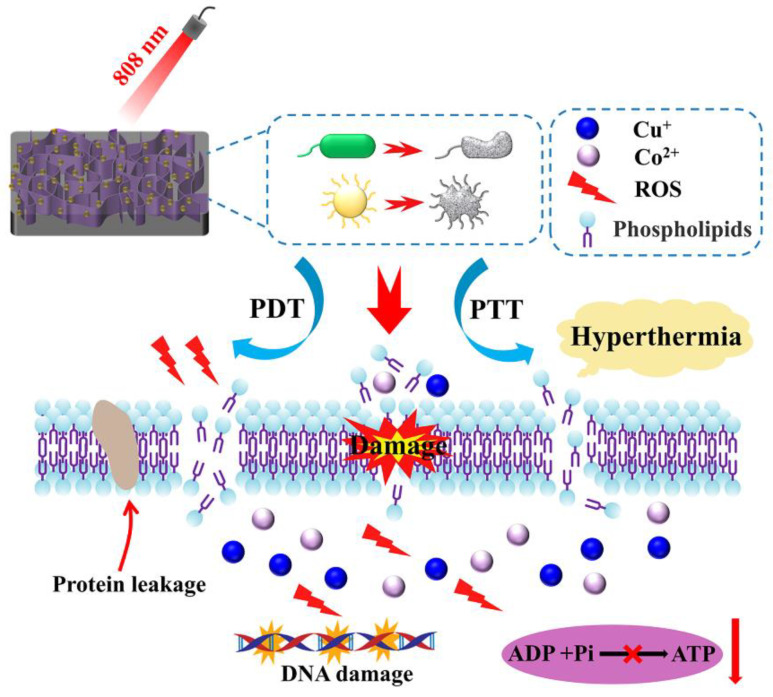
Schematic diagram for synergistic antibacterial mechanism.

According to the above results, a possible mechanism is proposed for PDA/Cu-O_v_-Co_3_O_4_@Ti in Figure 12. AFM topography images of bare Ti and PDA/Cu-O_v_-Co_3_O_4_@Ti are shown in Figure S11. The bare Ti shows a relatively smooth surface with an RMS value of 14.5 nm. In contrast, the RMS value of PDA/Cu-O_v_-Co_3_O_4_@Ti increased to 92.4 nm. It is seen that the smooth surface of bare Ti became rough after the modification of these interconnected nanosheets. In general, the rough surface was conducive to the bacterial adhesion and subsequent killing. Based on the above characterization, released metal ions, the photothermal effect, and the photodynamic effect will be involved in our antibacterial study.

Firstly, the released metal ions from substrates could lead to cytotoxicity towards bacteria. Moreover, the catechol structure in PDA could spontaneously undergo an oxidation reaction with O_2_ to produce the o-benzoquinone and H_2_O_2_ [61]. As proven by XPS, Cu^+^ ions existed on PDA/Cu-O_v_-Co_3_O_4_@Ti, which possessed Fenton-like activity with H_2_O_2_ for the generation of •OH. In particular, the Fenton-like activity could be further improved by the synergistic Cu−Co electronic coupling, which caused the upshift of the d-band center towards the Fermi level in Cu-O_v_-Co_3_O_4_; then, the dissociation of H_2_O_2_ into •OH was boosted due to the favorable electron donation to H_2_O_2_ [20]. The high electrochemical oxidation potential of •OH could be used effectively to result in both DNA damage and protein denaturation.

Secondly, the photothermal effect could cause physical damage to bacteria, inducing intracellular oxidative stress accompanied with the physical impedance with ultrathin nanosheets. In addition, the photothermal effect led to the enhanced membrane permeability for the invasion of metal ions or ROS, and the release of protein. In addition, such a mechano-bactericidal mechanism has been proven in a nanostructured surface by mimicking insect wings [62]. Meanwhile, the photothermal effect could promote the generation of •OH and ^1^O_2_, as confirmed by DST and DPBF probe tests, as well as EPR characterization upon NIR irradiation. All these factors cause severe oxidative stress on bacteria and then the metabolism was imbalanced, as confirmed by the downregulated ATP levels.

Thirdly, electron transition occurred from VB to CB in PDA/Cu-O_v_-Co_3_O_4_@Ti when excited by an 808 nm laser, resulting in the generation of electron-hole pairs. The high-energy electrons would reduce the o-benzoquinone structure during the generation of the H_2_O_2_ process, thus restoring the catechol structure. Meanwhile, type II energy transfer in photodynamic therapy could convert the dissolved O_2_ to ^1^O_2_, which has been proven by TEMP-trapped EPR tests. It is well concluded that the two types of ROS would efficiently inactivate the bacteria.

## 4. Conclusions

In summary, a multifunctional and highly biocompatible antibacterial nanocoating with medical Ti foils has been designed through H_2_ plasma treatment and PDA coating. By introducing O_v_- and Cu-doped atoms, the light absorption of the coating in the NIR region can be improved by 77.0%, resulting in an enhanced photothermal effect. The hyperthermia generated by NIR irradiation greatly improves the permeability of the bacterial outer and inner membranes, and the released Cu and Co ions and exogenous ROS can smoothly penetrate the bacterial membrane. At the same time, combined with the Fenton-like effect of Cu^+^, it can convert H_2_O_2_, supplied by the self-oxidation of PDA, into •OH. The antibacterial efficiency was about 100% for both Gram-negative and Gram-positive bacteria. Hyperthermia and physical impedance further stimulate bacteria to produce more endogenous ROS, disrupting their metabolic balance. Under the synergistic action of various functions mentioned above, the bacterial membrane ruptures, leading to protein leakage and a decrease in ATP levels, and resulting in its death. In addition, MTT results indicate that the viability of the L-929 cell is higher than 90%, indicating that PDA nanocoating has cell compatibility. This work proposes a promising route for multifunctional synergistic antibacterial medical Ti-based coatings to address issues such as post-implantation infections in the future.

## Data Availability

Data are contained within the article and Supplementary Materials.

## Supplementary Materials

The following supporting information can be downloaded at: https://www.mdpi.com/article/10.3390/ma17092019/s1, Figure S1: Digital and SEM images of (a,f) bare Ti foils, (b,g) Co_3_O_4_@Ti, (c,h) O_v_-Co_3_O_4_@Ti, (d,i) Cu-O_v_-Co_3_O_4_@Ti, and (e,j) PDA/Cu-O_v_-Co_3_O_4_@Ti; Figure S2: Raman spectra of PDA powder and PDA/Cu-O_v_-Co_3_O_4_@Ti; Figure S3: XPS survey scan of different samples including Co_3_O_4_@Ti, O_v_-Co_3_O_4_@Ti, Cu-O_v_-Co_3_O_4_@Ti, and PDA/Cu-O_v_-Co_3_O_4_@Ti; Figure S4: Water contact angles of bare Ti, Co_3_O_4_@Ti, O_v_-Co_3_O_4_@Ti, Cu-O_v_-Co_3_O_4_@Ti, and PDA/Cu-O_v_-Co_3_O_4_@Ti; Figure S5: Real-time thermal mapping corresponding to Co_3_O_4_@Ti, O_v_-Co_3_O_4_@Ti and Cu-O_v_-Co_3_O_4_@Ti during NIR irradiation for 10 min (808 nm, 1.5 W/cm^2^); Figure S6: Colony pictures corresponding to (a) *E. coli* and (b) *S. aureus* suspension in logarithmic phase stepwise dilutions during proliferation. Dynamic growth curves of (c) *E. coli* at 10 h and (d) *S. aureus* at 24 h in a 37 °C incubator; Figure S7: CLSM images of (a) *E. coli* and (b) *S. aureus*. Live bacteria were stained green with SYTO 9 and dead bacteria were stained red with PI; Figure S8: Zeta potentials of *E. coli*, *S. aureus*, Co_3_O_4_@Ti, O_v_-Co_3_O_4_@Ti, Cu-O_v_-Co_3_O_4_@Ti, and PDA/Cu-O_v_-Co_3_O_4_@Ti; Figure S9: Standard curves for protein leakage and ATP assay on bacteria; Figure S10: ICP tests corresponding to Cu and Co ions under different time; Figure S11: AFM topography images of (a) bare Ti and (b) PDA/Cu-O_v_-Co_3_O_4_@Ti.
