# Supplementary material for: Polydopamine-Coated Copper-Doped Co3O4 Nanosheets Rich in Oxygen Vacancy on Titanium and Multimodal Synergistic Antibacterial Study
Source: Materials (Basel). 2024 Apr 26;17(9):2019. doi: 10.3390/ma17092019 (PMC11084916; doi:10.3390/ma17092019)
Supplement: Supplementary file 1 [file materials-17-02019-s001.zip › materials-2970023-supplementary.pdf]

Supporting information

Polydopamine-coated Copper-doped Co_3O_4 Nanosheets Rich in Oxygen-Vacancy on Titanium and Multimodal Synergistic Antibacterial Study

Jinteng Qi[1], Miao Yu[2], Yi Liu[2], Junting Zhang[2], Xinyi Li[2], Zhuo Ma[3], Tiedong Sun[1], Shaoqin Liu[2] Yunfeng Qiu[2*]*

[a.] College of Chemistry, Chemical Engineering and Resource Utilization, Northeast Forestry University; Harbin, 150040, China.

[b.] Key Laboratory of Microsystems and Microstructures Manufacturing, School of Medicine and Health, Harbin Institute of Technology, Harbin 150080, China.

[c.] School of Life Science and Technology, Harbin Institute of Technology, Harbin 150001, China.

***Corresponding Author**

Prof. Tiedong Sun, E-mail: tiedongsun@nefu.edu.cn (Sun T)

Prof. Yunfeng Qiu, E-mail: qiuyf@hit.edu.cn (Qiu Y)

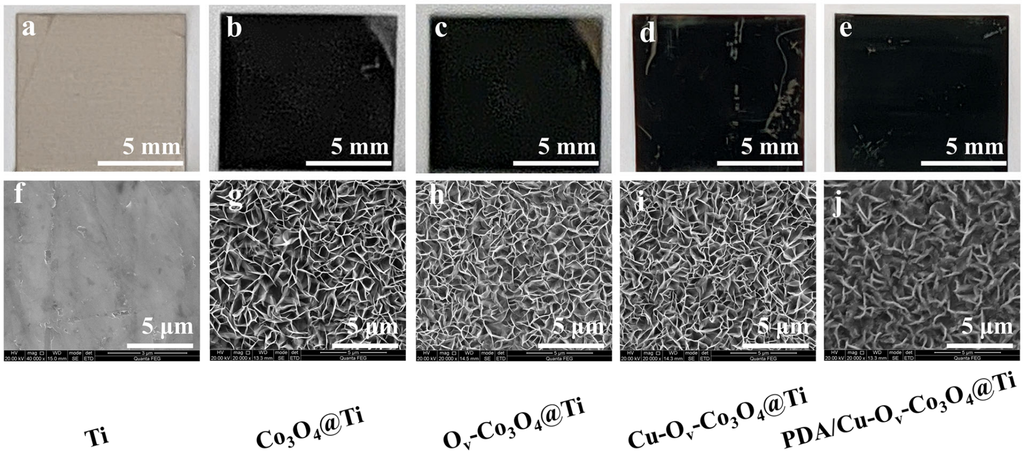

Fig. S1. Digital and SEM images of (a and f) bare Ti foils, (b and g) Co_3O_4@Ti, (c and h) O_v-Co_3O_4@Ti, (d and i) Cu-O_v-Co_3O_4@Ti, and (e and j) PDA/Cu-O_v-Co_3O_4@Ti.

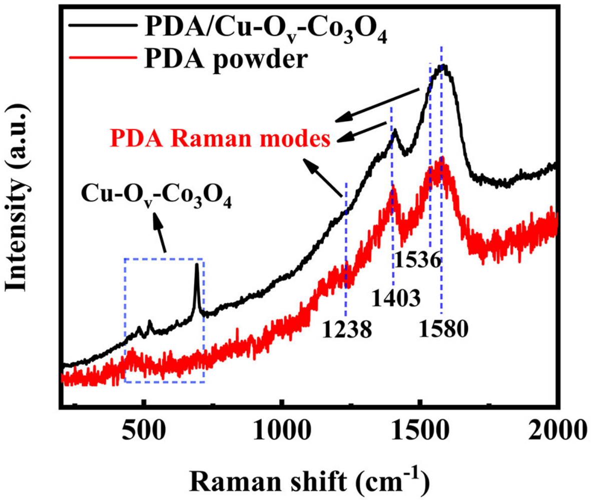

Fig. S2. Raman spectra of PDA powder and PDA/Cu-O_v-Co_3O_4@Ti.

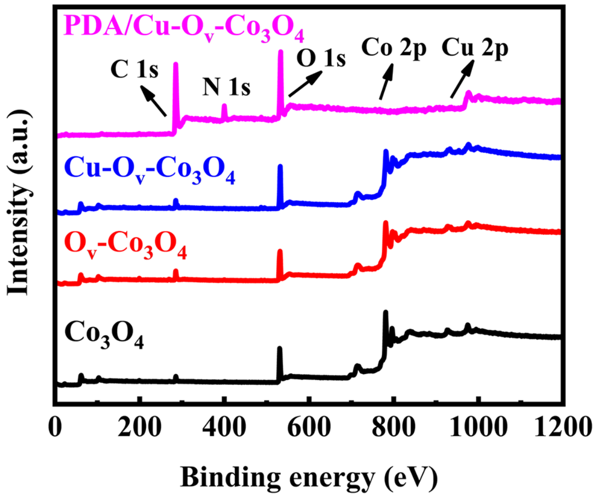

Fig. S3. XPS survey scan of different samples including Co_3O_4@Ti, O_v-Co_3O_4@Ti, Cu-O_v-Co_3O_4@Ti, and PDA/Cu-O_v-Co_3O_4@Ti.

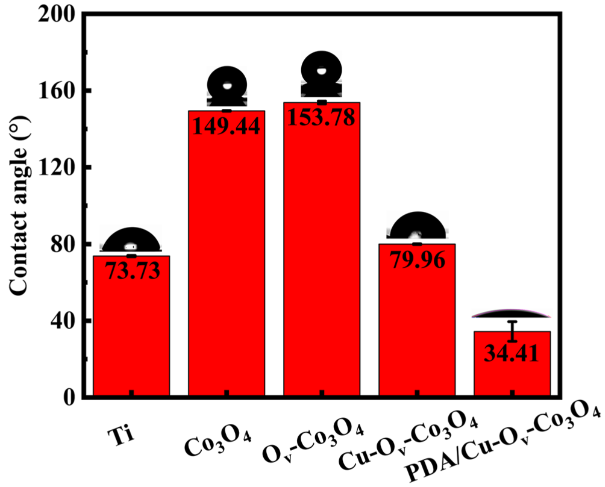

Fig. S4. Water contact angles of bare Ti, Co_3O_4@Ti, O_v-Co_3O_4@Ti, Cu-O_v-Co_3O_4@Ti, and PDA/Cu-O_v-Co_3O_4@Ti.

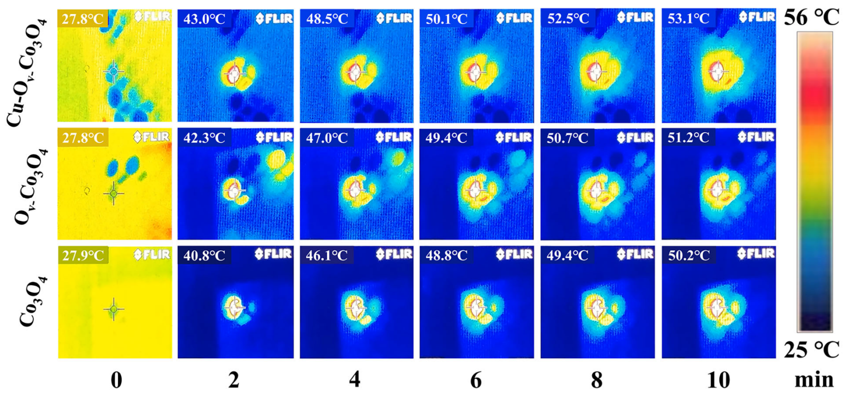

Fig. S5. Real-time thermal mapping corresponding to Co_3O_4@Ti, O_v-Co_3O_4@Ti and Cu-O_v-Co_3O_4@Ti during NIR irradiation for 10 min (808 nm, 1.5 W/cm^2).

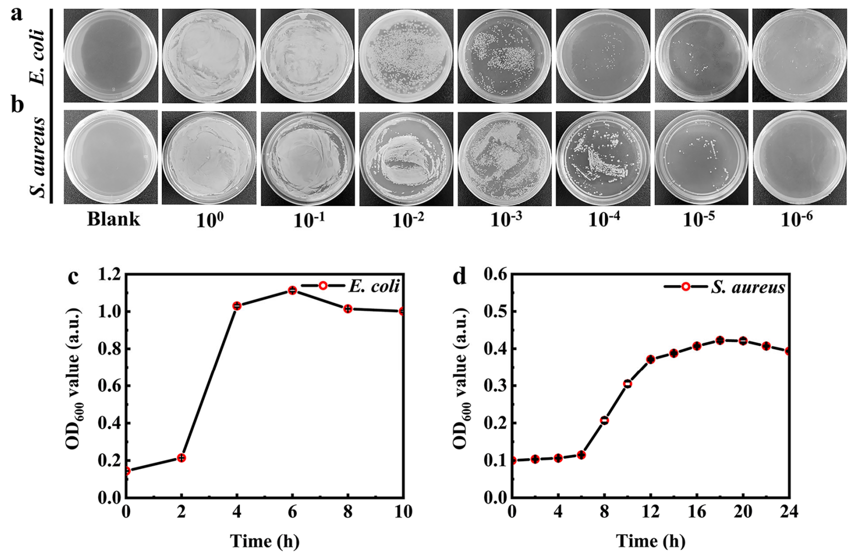

Fig. S6. Colony pictures corresponding to (a) *E. coli* and (b) *S. aureus* suspension in logarithmic phase stepwise dilutions during proliferation. Dynamic growth curves of (c) *E. coli* at 10 h and (d) *S. aureus* at 24 h in a 37 °C incubator.

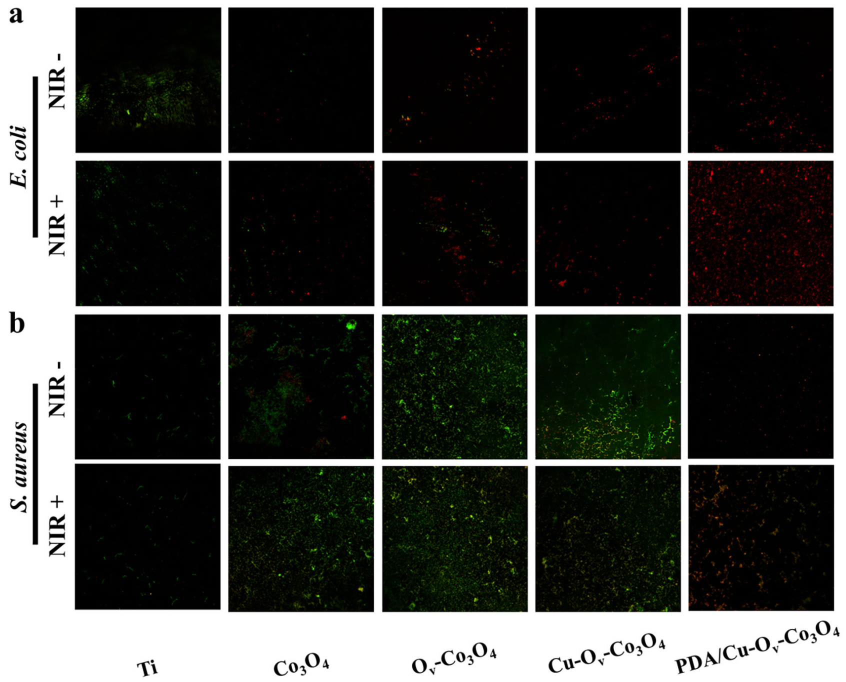

Fig. S7. CLSM images of (a) *E. coli* and (b) *S. aureus*. Live bacteria were stained green with SYTO 9 and dead bacteria were stained red with PI.

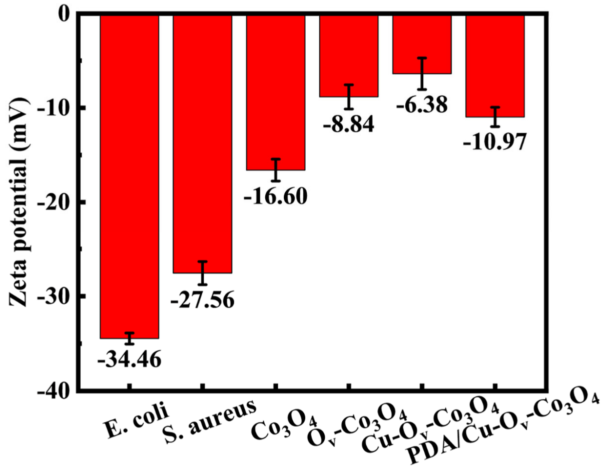

Fig. S8. Zeta potentials of *E. coli*, *S. aureus*, Co_3O_4@Ti, O_v-Co_3O_4@Ti, Cu-O_v-Co_3O_4@Ti, and PDA/Cu-O_v-Co_3O_4@Ti.

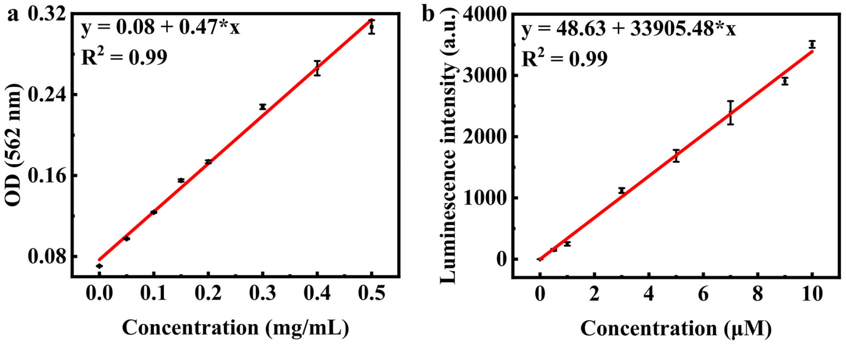

Fig. S9. Standard curves for protein leakage and ATP assay on bacteria.

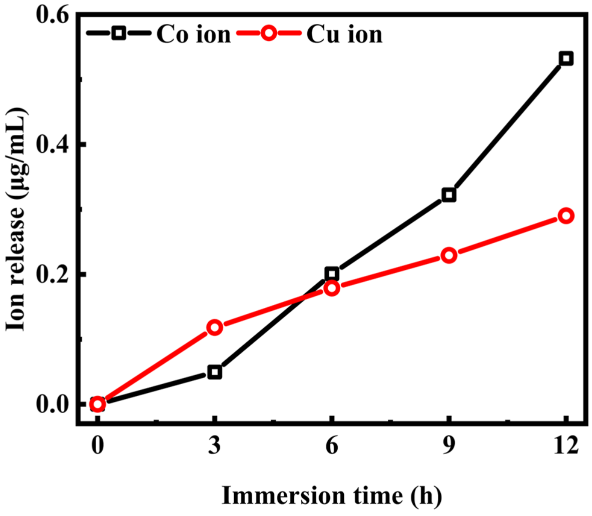

Fig. S10. ICP tests corresponding to Cu and Co ions under different time.

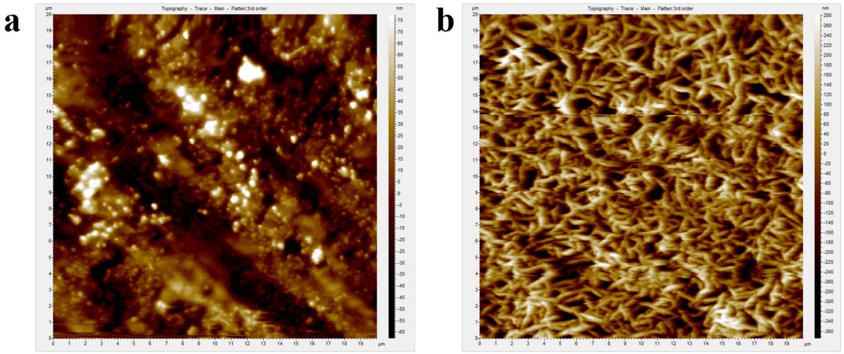

Fig. S11. AFM topography images of (a) bare Ti and (b) PDA/Cu-O_v-Co_3O_4@Ti.
